# Photobiomodulation therapy approach to a rare phenomenon of radiation recall reaction triggered by cisplatin in a cervical cancer patient: a case report and scoping review

**DOI:** 10.3389/fonc.2025.1700942

**Published:** 2026-02-02

**Authors:** Paloma Gomes, Wenzel Castro de Abreu, Ricardo Gomes dos Reis, Eliete Neves Silva Guerra, Elaine Barros Ferreira, Paula Elaine Diniz dos Reis

**Affiliations:** 1 Interdisciplinary Research Laboratory Applied to Clinical Practice in Oncology – LIONCO, Faculty of Health Sciences, University of Brasília, Brasília, Brazil; 2 Department of Radiation Oncology, University Hospital of Brasilia, Brasília, Brazil

**Keywords:** antineoplastic agents, case reports, low-level light therapy, radiation recall reaction, scoping review, uterine cervical neoplasms

## Abstract

**Purpose:**

Radiation recall reaction (RRR) is a rare and poorly understood phenomenon of tissue radiotoxicity. It is typically triggered by exposure to certain agents, most commonly cytotoxic drugs such as cisplatin. We conducted a scoping review following the methodology proposed by the JBI collaboration and PRISMA-ScR extension. It aimed to map the evidence concerning the treatment of RRR triggered by cisplatin.

**Methods:**

Searches were performed in PubMed/MEDLINE, EMBASE, Web of Science Core Collection, Cochrane Database, and ProQuest™ on March 9, 2025. Eligible studies included primary research, guidelines, and study protocols that addressed RRR as a primary or secondary outcome. A case report was also described.

**Results:**

Eight studies were included, and the main treatments for RRR were topical steroids and antihistamines. A 36-year-old woman with cervical cancer developed intense hyperpigmentation in the inguinal, vulvar, and anal areas, along with anal and labial fissures, dry desquamation in the inguinal region, and vaginal stenosis, 3 days after cisplatin. The diagnosis was RRR affecting mucosa and skin in the intimate region. Photobiomodulation therapy (PBMT) was initiated using lasers emitting wavelengths of 660 and 808 nm for tissue repair in the vulva, anus, and groin, and LEDs emitting 450 nm for vaginal stenosis and 590 nm for hyperpigmentation. Following the first PBMT session, the patient showed an excellent clinical response after 1 week, with a significant reduction in the severity of RRR.

**Conclusion:**

PBMT appears to be a viable, non-invasive, and low-cost treatment option for RRR, with no reported side effects.

## Introduction

In 2022, the global incidence of cervical cancer was 661,021 cases, representing 3.3% of all cancer diagnoses. Brazil accounted for 18,715 of these cases (1). More than 90% of women diagnosed with advanced cervical cancer require radiotherapy as part of their treatment. Chemotherapy, often involving platinum-based agents such as cisplatin, is commonly administered alongside RT (2, 3). During RT, healthy tissues are inevitably exposed to ionizing radiation. Even fractional doses of radiation can induce immediate structural damage, particularly in photosensitive organs such as the skin and mucous membranes (4).

An adverse effect of RT is the radiation recall reaction (RRR), a rare and poorly understood phenomenon of tissue radiotoxicity. First documented in 1959 as a skin manifestation, now known as cutaneous RRR (cRRR) or radiation recall dermatitis (RRD), this phenomenon has since been reported in multiple organs, including the lungs, stomach, vagina, and muscles (5–8).

RRR is typically characterized by an acute inflammatory response in areas previously exposed to RT. It is commonly triggered by cytotoxic agents, though recent reports also implicate targeted therapies, immunotherapy, hormone therapy, and even COVID-19 vaccines (6–11). While corticosteroids are frequently used for management, no standardized treatment protocol exists (7, 10, 12).

Given the absence of a standardized therapeutic approach, exploring non-invasive, painless, and cost-effective treatments with minimal adverse effects, such photobiomodulation therapy (PBMT), is crucial. PBMT can be used as an alternative or adjuvant to other treatment modalities, including pharmacotherapy (13). Therefore, the present study aims to map the evidence concerning the treatment of RRR cases in cancer patients previously undergoing RT triggered by cisplatin. Additionally, we also report a rare case of RRR affecting the skin and mucosa in a cervical cancer patient, triggered by cisplatin, and successfully managed with PBMT.

## Review of literature

A scoping review was conducted following the methodology proposed by the JBI collaboration (14). We utilized the Preferred Reporting Items for Systematic Reviews and Meta-Analyses (PRISMA) for Scoping Reviews (PRISMA-ScR) extension to report the results (15). The protocol has been registered on the Open Science Framework (OSF) and can be accessed via the Digital Object Identi"er (DOI) 10.17605/OSF.IO/SC6VZ (16).

### Eligibility criteria

The research question for this scoping review was formulated using the PCC acronym, which P refers to Population, C to Concept, and C to Context (14). The guiding question addressed here is: “What evidence exist in the literature regarding treatment of RRR (concept) in cancer patients previously undergoing radiotherapy (population) following exposure to a cisplatin agent (context)?”.

For this review, RRR is defined as an acute inflammatory response occurring in areas previously treated with radiotherapy, triggered by cisplatin, isolated or in association with other agents. Eligible studies included primary research, guidelines, and study protocols that addressed RRR as a primary or secondary outcome. Articles published in any language without time restrictions were included, ensuring a comprehensive literature search.

Exclusion criteria comprised: (1) studies involving patients without cancer; (2) studies involving patients who had not undergone radiotherapy; (3) studies not addressing RRR; (4) studies that did not inform the treatment for RRR; (5) reviews, book chapters, expert opinions, and conference abstracts; (6) *in vivo* or *in vitro* laboratories studies.

### Information sources and search strategy

An electronic literature search strategy was developed by researchers experienced in oncology and refined through team discussions. A tailored electronic search was conducted in PubMed/MEDLINE via the National Institutes of Health (NIH) and subsequently adapted for additional databases, including the EMBASE via Elsevier, Web of Science Core Collection (WoSCC) via Clarivate, and Cochrane Database via the Cochrane Library. Additionally, we searched gray literature using the ProQuest™ Dissertation & Theses Citation Index. Manual searches were performed on the reference lists of identified studies to uncover additional relevant literature. The complete search strategy is detailed in **Supplementary Material A**. All references were imported and organized using the software EndNote X9^®^ (Thomson Reuters, Philadelphia, PA, United States).

All searches were conducted on March 9, 2025. All articles were fully accessible online.

### Evidence screening and study selection

Duplicate records were removed using EndNote Basic and Rayyan^®^ (Qatar Computing Research Institute, Data Analytics, Doha, Qatar) (17), before the study selection, which occurred in two stages. In the first stage, two reviewers (PG and PEDR) independently screened citations from the search results, focusing on titles and abstracts. In the second stage, the selected studies were reviewed in full by two reviewers independently (PG and PEDR).

Eligibility was determined in both stages based on established inclusion and exclusion criteria. Articles not meeting the inclusion criteria were excluded. Any disagreements were resolved through discussion; intervention by a third reviewer (EBF) was not required.

### Data extraction process

Data extraction was conducted collaboratively by two reviewers (PG and PEDR). The extraction variables were determined through a thorough review of the articles, emphasizing the relevance of the information. Extracted data were organized in a Microsoft^®^ Excel 16.29.1 (Microsoft Office 2019, Microsoft, Redmond, United States) spreadsheet, where the reviewers mapped the findings, discussed results, and continually updated the data collection form. Consensus was sought for any disagreements.

### Data items

For descriptive data analysis, the reviewers collected a variety of items from the articles to ensure comprehensive understanding. Collected data included author(s), year of publication, country of the corresponding author, type of study, reported age of the sample, staging at diagnosis, tumor histology, neoadjuvant therapy, adjuvant therapy, use of antineoplastics concomitantly with radiotherapy, radiotherapy dose in Grays (Gy), radiotherapy fractions, time interval between the causative agent and RRR development, RRR area, RRR signs and symptoms, and treatment for RRR.

Based on the case description and available images, the authors graded the RRR severity using the Common Terminology Criteria for Adverse scale (CTCAE), version 5 (18).

### Synthesis of results

The extracted data from the included studies were grouped to synthesize the results descriptively in form of table.

## Results

**Figure 1:**
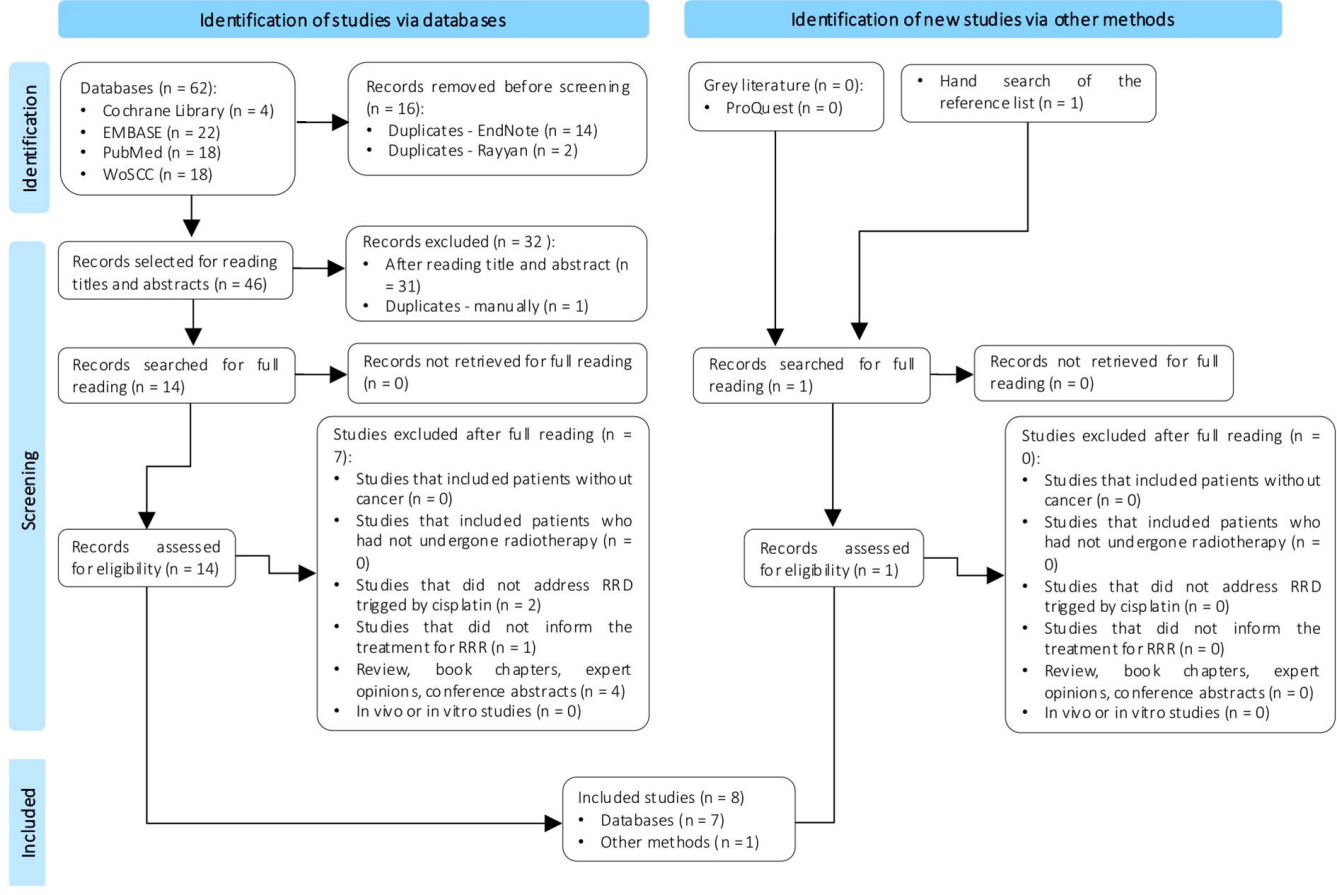
Flowchart for searching and selecting articles for the scoping review.

A total of 62 references were identified across four databases. After removing 16 duplicates, 46 studies proceeded to the first stage, which involved reviewing titles and abstracts. This resulted in the selection of 14 studies for full-text review, with seven ultimately being included. A total of seven studies were excluded because they did not meet the eligibility criteria (**Supplementary Material B**). Additionally, through other methods, including gray literature and manual searches of reference lists, one reference was identified (**Figure 1**). As a result, eight studies were included in this review.

This scoping review included eight studies (19–26), which collectively reported 14 cases of cancer patients. The types of cancer identified were anal carcinoma (19), lung carcinoma (20, 24), head and neck carcinoma (21, 22, 24, 25), cholangiocarcinoma (23), esophageal carcinoma (24), cervical carcinoma (26), lymphoma (24), and thymoma (24). The RRR presented in the form of radiodermatitis (19–25), vaginal inflammation (26), esophagitis (24), and mucositis (24). Only one patient (7.14%) exhibited two types of RRR, specifically RRD and mucositis (24). Cisplatin was administered either alone or in combination with other systemic antineoplastics, such as 5-fluorouracil (19), cyclophosphamide (24), cytarabine (24), gemcitabine (23), pemetrexed (24), pembrolizumab (24), rituximab (24), and vinorelbine (20). Seven patients (50%) (24) discontinued systemic antineoplastic treatment. The primary treatments for RRR included corticosteroids in nine patients (64.28%) (20, 22–24, 26), followed by antihistamines in seven patients (50%) (20, 22–24). One case (7.14%) (19) of RRR was treated with hyperbaric oxygen therapy due to the presence of an ulcerative lesion. To date, there have been no reports of RRR treatment using PBMT. A summary of the results is provided in **Table 1**.

**Table 1:** Summary of results of eight studies included.

Author, year, country	Clinical	Antineoplastic treatment	Time CDDP-RRR	Type and severity of RRR	Clinical type of RRR	RRR treatment
Sroa et al. (19) 2006 Case report and a review of literature United States	47-year-old female, with squamous cell carcinoma of the anus, T4N3M0, stage IIIB	Neoadjuvant therapy—CT+RT: two cycles of 5-FU and mitomycin C; intraoperative high-dose radiation brachytherapy directed to her perineum was administered as a single dose of 10 Gy; external beam RT was directed to her pelvis using the anteroposterior and posteroanterior fields with mixed 6 and 18 MV photons, representing a total dose of 45 Gy in 25 fractions. Following this, a rectal boost was applied with 18 MV photons, providing an additional dose of 5.14 Gy in three fractions over 4 days. Concurrently, RT with 15-MeV electrons was directed at the bilateral inguinal lymph node chains, delivering a dose of 5.4 Gy in three fractions Adjuvant therapy—two cycles of 5-FU at 1,000 mg/m^2^ for 4 days and CDDP at 75 mg/m^2^ for 1 day	3 months after completion of the second course of 5-FU and CDDP	RRD grade 4 (CTCAE version 5)—skin necrosis and ulceration of full thickness dermis	The patient developed tender, erythematous, and indurated perianal plaques with a symmetrical distribution bilaterally on the buttocks, closely corresponding to one of the RT fields. These plaques have persisted, and the pain associated with the extensive areas of induration has become significant, precluding any direct pressure to the area	Hyperbaric oxygen therapy to alleviate the inflammation
Zhu et al. (20) 2010 Case report China	59-year-old female, with lung adenocarcinoma, T2N3M0, stage IIIB	Neoadjuvant therapy—CT+RT: two courses of 40 mg of vinorelbine (25 mg/m^2^) on days 1 and 8, in combination with 40 mg of CDDP (25 mg/m^2^) from day 1 to day 3 of each course; 3DCRT using 6−MV photons with four beams, targeting both the lung tumor and the metastatic left supraclavicular lymph nodes. The patient received a total dose of 65 Gy in 25 fractions, with 2.5 Gy/fraction for the first 20 fractions, followed by 3 Gy/fraction for the subsequent five fractions. Adjuvant therapy—isolated CT: 40 mg of vinorelbine (25 mg/m^2^) on days 1 and 8, in combination with 40 mg of CDDP (25 mg/m^2^) from day 1 to day 3	22 days after vinorelbine and CDDP	RRD grade 2 (CTCAE version 5)—moderate to brisk erythema	The patient experienced intense itching in the irradiated regions of the prothorax and the left supraclavicular area. Physical examination revealed noticeable redness and swelling of the local skin, characterized by extensive erythema covering the irradiated field, without skin ulceration	Oral clarityne and local administration of chloramphenicol dexamethasone for 1 week. The symptoms completely disappeared after 3 weeks
Melnyk et al. (21) 2012 Case report United States	49-year-old male, squamous cell carcinoma of the tonsil, T2N1M0, stage III	Adjuvant therapy—CT+RT: CDDP and daily RT was initiated. The tumor bed and ipsilateral neck were treated with 2-Gy fractions to 70 Gy, while the contralateral neck received 1.7-Gy fractions to 56 Gy. The CDDP was administered at a dose of 100 mg/m^2^ on days 2, 22, and 43 of his 6 weeks of RT	Approximately 9 months after his last radiation or CT treatment	RRD grade 2 (CTCAE version 5)—moderate to brisk erythema	The patient presented with a 24-h history of a tender, hot, confluent rash on the right side of his neck, beginning at the clavicle and extended superiorly to the mandibular angle. He reported an oral temperature of 102° F, along with myalgias and arthralgias	Due to the warmth of the tissue and the fever, there was some concern for cellulitis, leading to treatment with doxycycline. The patient reported that his rash resolved in the 5 to 6 days following the initial presentation
Kindts et al. (22) 2014 Case report Belgium	68-year-old female, undifferentiated non-keratinizing invasive squamous cell carcinoma of the nasopharynx T2N2cM0, stage III	Neoadjuvant therapy—CT+RT: 1-week cycles of CDDP. For RT, 70 Gy was given to the tumor and affected lymph nodes and 50 Gy to the bilateral retropharyngeal, submental, submandibular, upper, middle, and lower internal jugular and spinal accessory lymph nodes, in daily fractions of 2 Gy using external beam IMRT, 6-MV beams	13 days after the last cycle of CT	RRD grade 2 (CTCAE version 5)—moderate to brisk erythema	The patient exhibited extensive, tender, bilateral erythema and micropapular eruptions at the previously irradiated skin. This painful inflammatory erythema was confined exclusively to the areas that had received radiation	Oral methylprednisolone (32 mg) was administered for one week, in combination with ebastine and local application of methylprednisolone. After 7 days, the RRD had disappeared, allowing for the gradual discontinuation of methylprednisolone
Bahaj et al. (23) 2019 Case report United States	79-year-old man with a history of right arm soft-tissue tumor of unknown etiology, and a second primary intrahepatic cholangiocarcinoma	Surgical resection followed by RT (an unknown dose) to the right arm and shoulder area for the soft-tissue tumor in 1951 In 2017, the patient was treated with a combination of simvastatin, gemcitabine (1 g/m^2^), and CDDP (25 mg/m^2^)	Four days after cycle 1	RRD grade 2 (CTCAE version 5)—moderate to brisk erythema	The patient developed an erythematous, coalescent patchy rash on the right shoulder and arm, which extended to the chest wall. This rash was geographically confined to the site of previous RT for a soft-tissue tumor	He was managed symptomatically with topical triamcinolone and oral antihistamines. The rash improved and completely resolved within 3 weeks. Upon resolution of the dermatitis, the patient was rechallenged with the same dose of gemcitabine/CDDP, which he tolerated well this time
Purkayastha et al. (24) 2022 Prospective observational study India	Non-small cell lung cancer	60 Gy and CDDP 75 mg/m^2^ + pemetrexed 500 mg/m^2^	3–5 weeks after CT	RRD	NI	Discontinuation of CT + normal saline compresses + aqueous based cream + topical steroid 0.1% mometasone furoate cream/0.1% betamethasone cream + antihistamines
	Carcinoma lung with brain metastasis	30 Gy and CDDP 75 mg/m^2^ + pemetrexed 500 mg/m^2^ + pembrolizumab 200 mg	5 weeks after CT	RRD	NI	Discontinuation of CT + normal saline compresses + aqueous based cream + 10% glycerine + topical steroid 0.1% mometasone furoate cream/1% hydrocortisone cream + gentian violet dressing + oral NSAIDs
	Carcinoma nasopharynx	70 Gy and CDDP 75 mg/m^2^ + 5−FU 800 mg/m^2^ (3 cycles)	3–5 weeks after CT	Mucositis	NI	Discontinuation of CT + soda−saline gargles + 2% oral viscous lidocaine + analgesic benzydamine hydrochloride
			3–5 weeks after CT	RRD	NI	Discontinuation of CT + normal saline compresses + aqueous based cream + topical steroid 0.1% mometasone furoate cream/0.1% betamethasone cream + antihistamines
	Adenocarcinoma esophagus	41.4-50.4 Gy and 5−FU 2,000 mg/m^2^ + CDDP 50 mg/m^2^ (4 cycles)	3–5 weeks after CT	RRD	NI	Discontinuation of CT + normal saline compresses + aqueous based cream + topical steroid 0.1% mometasone furoate cream/0.1% betamethasone cream + antihistamines
	Relapsed diffuse large B-cell lymphoma	36 Gy and dexamethasone 40 mg + cytarabine 2,000 mg/m^2^ + CDDP 100 mg/m^2^ + rituximab 375 mg/m^2^ (3 cycles)	3–5 weeks after CT	RRD	NI	Discontinuation of CT + normal saline compresses + aqueous based cream + topical steroid 0.1% mometasone furoate cream/0.1% betamethasone cream + antihistamines
	Refractory classical Hodgkin lymphoma	36 Gy and dexamethasone 40 mg + cytarabine 2 g/m^2^ + CDDP 100 mg/m^2^ (2 cycles)	> 5 weeks after CT	Esophagitis	NI	Discontinuation of CT + avoiding potentially irritant foods + syrup sucralfate + 2% oral viscous lidocaine + oral proton pump inhibitors
	Recurrent thymoma	54 Gy and CDDP 50 mg/m^2^ + doxorubicin 50 mg/m^2^ + cyclophosphamide 500 mg/m^2^ (2 cycles)	3–4 weeks after CT	RRD	NI	Discontinuation of CT + normal saline compresses + aqueous based cream + topical steroid 0.1% mometasone furoate cream/0.1% betamethasone cream + antihistamines
Tamaskovics et al. (25) 2023 Case report and focused systematic review Germany	38-year-old female, with an *in situ* adenocarcinoma of the left nasal cavity and the left paranasal sinuses and locally advanced recurrence of an adenocarcinoma (G3) in the left middle ear, Eustachian tube, and nasopharynx, without any distal metastases	Adjuvant therapy—local RT with IMRT, 50.4 Gy in 28 fractions, in the left nasal cavity and the left paranasal sinuses. Due to metastases from a high-grade adenocarcinoma in multiple lymph nodes with extracapsular extension, a bilateral neck dissection was performed followed by RT with IMAT, 66 Gy in 33 fractions. Two cycles of CDDP 20 mg/m^2^ and day, d1–5 and 5-FU 600 mg/m^2^ and day, d1–5, continuous infusion was given parallel to RT Postoperative re-irradiation with IMAT, 60 Gy in 30 fractions, in combination with weekly CDDP 40 mg/m^2^	6 h after the first administration of CT	RRD grade 2 (CTCAE version 5)—moderate to brisk erythema	The patient developed an erythema of the neck (CTCAE grade 1) along with dysesthesia (CTCAE grade 1). The sharp border of the inflammatory skin reaction corresponded to the site of bilateral cervical lymph node irradiation performed a year earlier, roughly aligned with the 20-Gy isodose at the skin surface. Mild RRD recurred after each of the five subsequent CDDP, with decrescendo kinetics	The RRR resolved within 3 days with topical therapy using panthenol and moisturizing creams containing alpha-linolenic acid. The treatment protocol remained unchanged. Mild RRD recurred after each of the five subsequent doses of CDDP, with decrescendo kinetics
Carvalho et al. (26) 2023 Case report Brazil	47-year-old female, diagnosed with a bulky cervical adenocarcinoma, 6 cm size, stage IIB	Neoadjuvant therapy - CT+RT: CDDP 50 mg/m^2^; external-beam RT using the IMRT + IGRT, with a target volume of 50Gy in 25 fractions included the uterus, the parametria, and the upper one-third of the vagina. The target volume of 45Gy in 25 fractions included common, external, internal/obturator, and presacral iliac nodes. As for brachytherapy, the patient was given four insertions of Fletcher-type applicators, with a dose of 7 Gy being prescribed at point A in each of the insertions. The patient underwent laparoscopic completion hysterectomy plus bilateral salpingo-ophorectomy Adjuvant therapy: because of residual disease in the hysterectomy specimen, the patient was submitted to additional 3 cycles of CDDP 50 mg/m^2^ D1 and gemcitabine 1 g/m^2^ D1 and D8	< 4 months	Vaginal inflammation grade 4 (CTCAE version 5)—life-threatening consequences; widespread areas of mucosal ulceration; urgent intervention indicated	The patient presented with bleeding from the vaginal dome. Gynecological examination showed a mass with a necrotic appearance, suggestive of recurrence. Magnetic resonance imaging revealed a solid, retracted lesion measuring 6.2 × 2.2 × 3.0 cm adjacent to the vaginal dome. The lesion extended bilaterally, involving the distal segments of both ureters and causing moderate upstream ureterohydronephrosis	Dexamethasone 20 mg/day for 4 months

3DCRT, three-dimensional conformal radiotherapy; 5−FU, 5-fluorouracil; CDDP, cisplatin; CT+RT, chemoradiotherapy; CT, chemotherapy; CHOP, cyclophosphamide, doxorubicin, vincristine, prednisolone; CTCAE, Common Terminology Criteria for Adverse Events; FAC, 5-fluorouracil, doxorubicin, and cyclophosphamide; Gy, Grays; IGRT, image-guided radiation Therapy; IMAT, intensity-modulated arc therapy; IMRT, intensity-modulated radiation therapy; RRD, radiation recall dermatitis; RT, radiotherapy.

## Case report

A 36-year-old Brazilian woman with Fitzpatrick skin phototype IV was diagnosed with T1b3N0M0, grade 1 squamous cell carcinoma of the uterine cervix. Her medical history included hypertension, managed with propranolol (10 mg daily), and a 15-year history of smoking (10 cigarettes per day), social drinker. She reported no active sex life or use of contraceptive methods. The patient had no known allergies.

**Figure 2:**
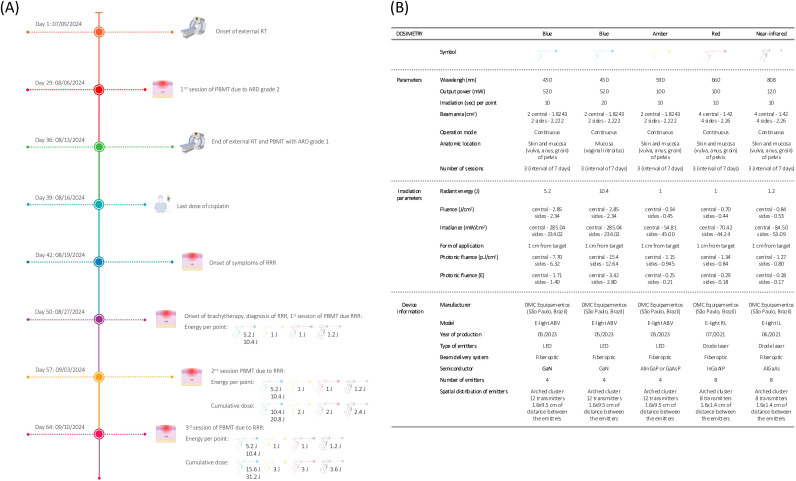
Patient timeline and dosimetry of PBMT. **(A)** Patient timeline from the start of RT to the last PBMT session. **(B)** Dosimetry of PBMT. RT, radiotherapy; PBMT, photobiomodulation therapy; ARD, acute radiodermatitis; RRR, radiation recall reaction; nm, nanometer; mW, milliwatt; sec, second; cm^2^, square centimeter; J, Joule; J/cm^2^, Joule per square centimeter; mW/cm^2^, milliwatt per square centimeter; p·J/cm^2^, photonic Joule per square centimeter; E, Einstein; LED, light-emitting diode. 1 Einstein = 4.5 p·J/cm^2^ which is the photonic fluence at 810 nm that is equivalent to the conventional fluence of 3 J/cm^2^. Photon energy at 450 nm = 2.7 eV; 590 nm = 2.1 eV; 660 nm = 1.9 eV; 808 nm = 1.5 eV.

Adjuvant antineoplastic treatment was initiated with three-dimensional conformal RT (3D-CRT) using a Varian^®^ Clinac linear accelerator, model CX (Palo Alto, CA, USA). The treatment consisted of a total dose of 50 Grays (Gy) delivered in 25 fractions (2 Gy per day) to the pelvic region using four static 10-megavolts (MV) photon beams, combined with weekly chemotherapy using cisplatin at a dose of 40 mg/m². RT was initiated on 9 July 2024 (day 1 in **Figure 2A**). The patient was treated in a supine position. Daily positioning was performed using orthogonal radiographs.

During the 20th RT session (day 29 in **Figure 2A**), after a cumulative dose of 40 Gy, the patient developed grade 2 acute radiodermatitis (ARD) according to CTCAE version 5 criteria (18). Treatment with PBMT was then initiated using low-dose light from lasers and light-emitting diodes (LEDs).

On gynecological examination, the patient presented intense hyperpigmentation in the vulvar, inguinal, and anal regions, along with edema in the labia minora and majora, as well as fissures in the labia minora and anus. The patient reported pain and burning sensation in the genital region, particularly following spontaneous diuresis. PBMT was combined with a cooling gel mask (Cool Care Mask WeCare^®^) to alleviate the burning symptoms. Within 72 h of initiating PBMT, the patient exhibited excellent clinical improvement, with complete resolution of edema, microfissures, and pain.

PBMT and the gel mask were continued at 72-h intervals between RT sessions, combining laser (red and near-infrared) and LED (blue and amber) treatments. By the end of RT (day 36 in **Figure 2A**), the patient showed grade 1 ARD (CTCAE version 5) (18), with only moderate residual hyperpigmentation.

The final dose of cisplatin was administered 3 days after the completion of RT (day 39 in **Figure 2A**). However, within 3 days of cisplatin administration (day 42 in **Figure 2A**), the patient experienced a sudden worsening of symptoms, including increased hyperpigmentation and the reappearance of anal and labial fissures, as well as vaginal stenosis.

The patient returned to the service to undergo brachytherapy 11 days after the last dose of cisplatin (day 50 in **Figure 2A**). The brachytherapy was applied using an Iridium-192 source, administered 24 Gy in three fractions with an interval of 7 days. Upon clinical evaluation, intense hyperpigmentation was noted in the inguinal, vulvar, and anal regions, along with anal and labial fissures, dry desquamation in the inguinal area. The patient reported narrowing of the vaginal canal. Assessment of vaginal stenosis was not performed on that same day, as she had already undergone brachytherapy and the examination would have caused additional discomfort. Based on her clinical history and presentation, the patient was diagnosed with RRR affecting the skin (grade 1—CTCAE version 5.0 (18)) of the inguinal region and vaginal and anal mucosa.

As this is the first reported case of RRR treated with PBMT and no clinical guidelines currently recommend the use of PBMT for this condition, we followed the recommendations provided in the WALT position paper (27) for ARD to guide the treatment of RRR, given the similarities between the two conditions.

According to this document, WALT recommends transcutaneous PBMT using LED or laser devices emitting wavelengths in the visible spectrum, ranging from violet, blue, green, amber, and red light, to near-infrared light (400-1,100 nm). Treatments should be repeated until a clinical benefit is observed (27). The rationale for the specific multi-wavelength PBMT protocol (450, 590, 660, and 808 nm) was based on the known biological effects of each wavelength on the targeted tissues and symptoms observed in the patient.

Red and near-infrared lights are known to modulate the inflammatory process, promote tissue repair, reduce edema, and alleviate pain (28), supporting the rationale for using these wavelengths in the treatment of RRR. Amber light inhibits melanosome maturation and reduces melanin content and tyrosinase activity (29), supporting the rationale for using amber PBMT as an adjuvant approach for the treatment of cutaneous hyperpigmentation in patients with RRR. Blue light has been used in the management of vaginal stenosis in patients undergoing radiotherapy due to its potential to address mucosal alterations, as blue LED exhibits tissue-reparative effects (30). These findings provide the rationale for using blue PBMT as an adjuvant approach to improve vaginal stenosis in patients with RRR, although vaginal stenosis was not an outcome measured in this case.

Therefore, PBMT was reinitiated using laser with wavelengths of 660 nanometers (nm) (red) at 1 Joule (J) of energy per point for 10 s for tissue repair in the vulva, anus, and groin and laser with wavelengths of 808 nm (near-infrared) at 1.2 J per point for 10 s for tissue repair in the same areas. Due to the patient’s intolerance to contact mode because of the heating sensation, the laser device was positioned less than 1 cm from the skin during treatment. Additionally, LEDs with a wavelength of 450 nm (blue) were applied at 5.2 J per point for 10 s for tissue repair in the vulva, anus, and groin, and 10.4 J per point for 20 s for vaginal stenosis at the vaginal introitus. LEDs with a wavelength of 590 nm (amber) were used at 1 J per point for 10 s for hyperpigmentation in the vulva, anus, and groin. Based on the WALT position paper (27) and Esteves-Pereira et al. (31), the PBMT parameters are described in **Figure 2B**.

**Figure 3:**
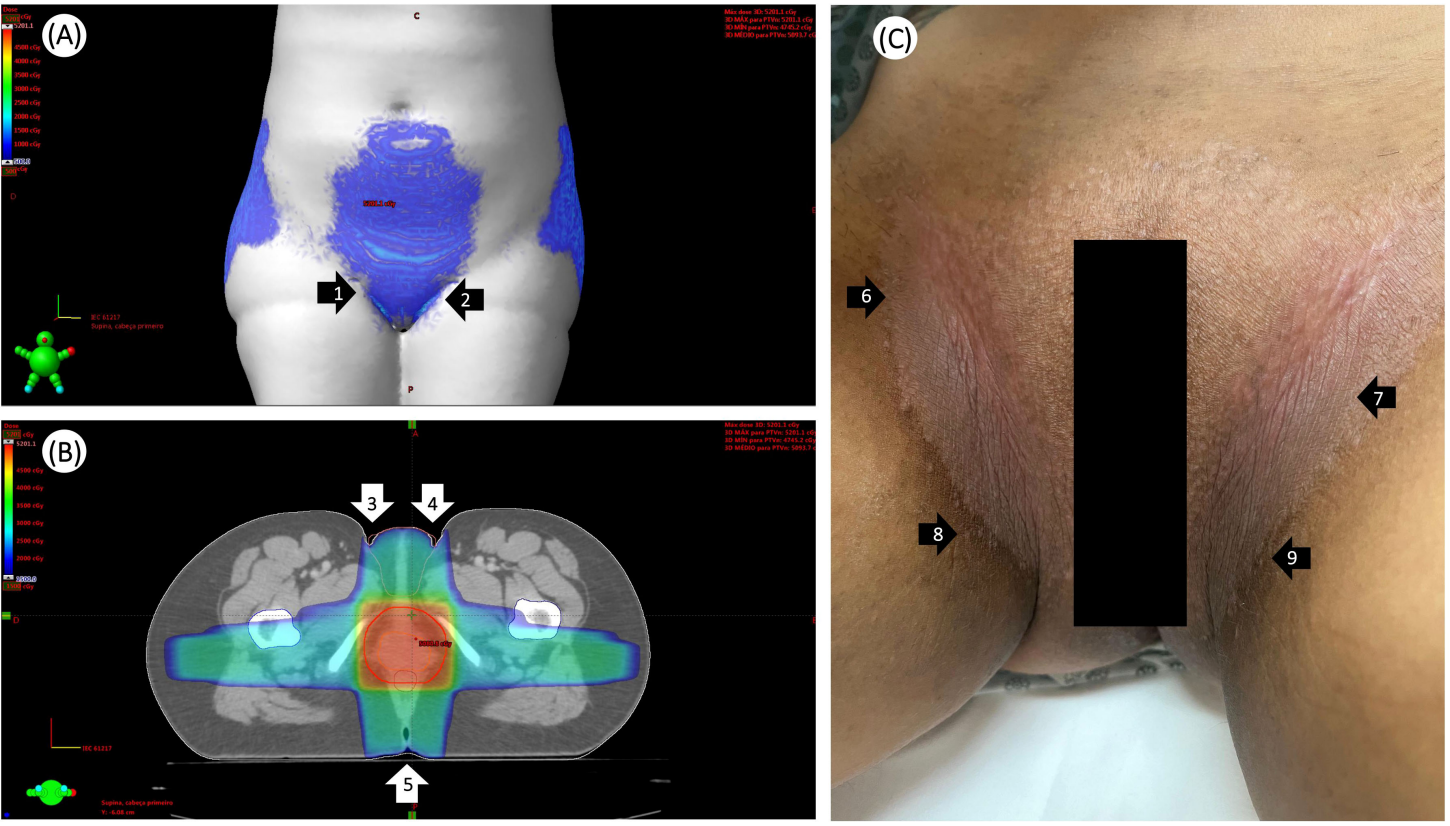
Radiation field to the pelvis delivered and completed on August 13, 2024. **(A)** Doses ranging from 5 Gy (dark blue) to 20 Gy (light blue) on the patient’s skin surface, according to the Treatment Planning System (TPS). Arrows 1 and 2 indicate the fold regions on the skin surface that received higher doses. **(B)** Shows the dose distribution in a color wash of a four-field treatment plan in an axial slice. Arrows 3, 4, and 5 indicate the folds in the inguinal and intergluteal regions. **(C)** Arrow 6, area of dry desquamation in the right inguinal region fully healed after the 1st session of photobiomodulation therapy (PBMT); arrow 7, areas of dry desquamation in the left inguinal region fully healed after 1st; arrow 8, area of hyperpigmentation in the right inguinal region persisted after 1st session of PBMT; arrow 9, area of hyperpigmentation in the left inguinal region persisted after 1st session of PBMT.

Seven days (day 57 in **Figure 2A**) after the first PBMT session, the patient showed an excellent clinical response, with a notable reduction in the severity of RRR. While intense hyperpigmentation in the inguinal, vulvar, and anal areas, as well as vaginal stenosis, persisted, the anal and labial fissures and dry desquamation in the inguinal region had fully healed (**Figure 3**).

During the second and third PBMT sessions, the same laser and LED parameters were maintained. Flamigel RT^®^ 100 mg was used concurrently with all PBMT sessions to relieve symptoms. By the end of PBMT protocol, 14 days (day 64 in **Figure 2A**) after the first session of PBMT, the patient exhibited only moderate hyperpigmentation in the inguinal, vulvar, and anal regions, with persistent vaginal stenosis. **Figure 2A** illustrates the patient’s treatment timeline. **Figure 2B** provides the dosimetric parameters used in PBMT.

## Discussion

To the best of our knowledge, this is the first case report of RRR treated with PBMT. The aim of RRR treatment is to alleviate symptoms and provide supportive care, with the approach varying depending on the severity of the reaction and the organs involved. In severe cases, the first step is often to discontinue the drug that triggered the reaction (7). In this scoping review, half of the patients discontinued systemic antineoplastic treatment (24) and the majority of cases were treated with systemic and/or topical corticosteroids.

Until now, corticosteroids are primarily used as supportive treatment for cancer-related complications and side effects of antineoplastic treatments, such as radiotherapy. Diabetes mellitus, poorly controlled arterial hypertension, recent acute coronary syndrome, peptic ulcer disease, and osteoporosis represent relative contraindications to corticosteroid treatment (32). It is worth noting that a significant portion of cancer patients have a second associated comorbidity (33). The risk of adverse events associated with corticosteroids increases with cumulative dosage and treatment duration, including disorder related to glycolipid metabolism and disorders related to the endocrine, hematologic/immunologic, neuropsychological, musculoskeletal, cardiovascular, and dermatological systems. Adverse events in the skin include lipodystrophy, skin atrophy, erosions, striae rubrae, ecchymosis, as well as acne, hirsutism, and hair loss (32).

Considering the multiplicity of significant adverse events that corticosteroid administration can cause, and the misuse of corticosteroids, this study also describes a case in which PBMT was used to treat RRR in the skin and mucosa, triggered by the final dose of cisplatin in a patient with cervical cancer. PBMT can be a safe, non-invasive, low-cost, non-thermal treatment, with no side effects reported to date (34).

PBMT involves the targeted application of low-power light sources, such as lasers and LEDs, to stimulate healing, relieve pain, and modulate the inflammatory and healing process (28). In recent years, PBMT has shown a favorable safety profile and positive clinical outcomes for cancer patients (34–36). There is substantial evidence supporting the use of PBMT for preventing and treating a wide range of complications related to cancer treatment, including radiodermatitis, oral mucositis after chemotherapy and radiotherapy, osteoradionecrosis, mucosal necrosis, oral and dermatologic chronic graft-versus-host disease, and radiation fibrosis (27, 35, 37).

Although the specific cellular signaling mechanisms behind PBMT are not yet fully understood, it is believed that its effects are primarily due to a decrease in reactive oxygen species (ROS) in cells and tissues under oxidative stress. Other potential mechanisms include the involvement of nitric oxide (NO), cyclic adenosine monophosphate (cAMP), and calcium signaling pathways. The chromophores responsible for PBMT’s effects include cytochrome C oxidase (CCO), light-gated ion channels and opsins, flavins, and flavoproteins, as well as water and heat-sensitive ion channels, particularly from the mitochondria in eukaryotic cells (28, 38).

The prevention and management of RT-induced adverse effects on the skin using PBMT have been investigated in reviews and clinical trials involving cancer patients with ARD (39–43). These studies suggest that PBMT reduces the severity of ARD and improves patients’ quality of life during RT (42, 43). A systematic review with meta-analysis concluded that PBMT is effective in preventing ARD, particularly grade 3, and in reducing pain (39).

In this present case, PBMT also induced regression in the severity of cutaneous RRR and, according to the patient’s report, provided pain relief and improved her quality of life. Since RRR is an acute inflammatory response in areas previously exposed to RT (5, 11), PBMT may modulate the inflammatory process and promote tissue repair of the skin and mucosa, as well as provide pain relief (28).

The mechanisms by which PBMT may have mitigated RRR in the patient reported in this case report may be closely related to its modulatory effects on mitochondrial activity and inflammatory pathways activated by cisplatin administration. Photon absorption by CCO may have induced photodissociation of inhibitory NO, thereby enhancing CCO activity, oxygen consumption, and adenosine triphosphate (ATP) synthesis in the mitochondria. Additionally, PBMT reduces ROS levels in oxidatively stressed or inflamed tissues, such as those affected in RRR, through the upregulation of antioxidant defenses, downregulation of NF-κB activation in inflamed cells, reduction of M1 macrophage markers, and decreased reactive nitrogen species and prostaglandins (28).

Modulate the inflammatory process and tissue-repair effects of PBMT have also been studied in mucous membranes. A case report described the use of PBMT to treat vulvovaginal symptoms induced by graft-versus-host disease. In that case, the application of 660- and 808-nm laser (100 mW, 4 J, twice a week) promoted pain relief and reduced burning sensation and discomfort, likely due to PBMT’s anti-inflammatory properties and tissue repair capabilities, resulting in significant improvement in daily activities (44).

Additionally, a clinical trial found that the use of a 401-nm blue LED on healthy vaginal mucosa did not induce pathogenic changes in vagina, suggesting that blue LED may be a potential alternative for treating vulvovaginal dysfunctions such as vulvovaginal candidiasis, bacterial vaginosis, and vulvovaginal atrophy, due to its antimicrobial and regenerative properties (45). A case report involving two women who underwent intravaginal and extravaginal treatment with 405-nm blue LED demonstrated promising tolerance and potential as a treatment option to vaginal stenosis following pelvic RT (30).

However, in our case, the patient was still undergoing pelvic brachytherapy and only received treatment with extravaginal blue LED; thus, she did not report an improvement in vaginal stenosis. The lack of reported improvement in vaginal stenosis may be attributable to several factors: the ongoing brachytherapy, which continues to induce tissue changes; the exclusive use of extravaginal application rather than a combination with intravaginal application; the relatively short follow-up period; or an intrinsic limitation of PBMT in reversing established fibrotic changes in the vaginal canal. This observation highlights the need for further studies to clarify the optimal timing, route of application, and efficacy of PBMT for radiation-induced vaginal stenosis.

An *in vitro* study that investigated the effects of 585-nm amber LED on melanogenesis in cultured human epidermal melanocytes (HEMs) found that this wavelength negatively regulates melanin production in human melanocytic cells by inhibiting melanogenesis in a dose-dependent manner and by stimulating autophagy processes (29). In our case, the patient exhibited attenuation of hyperpigmentation in the genital area after three PBMT sessions.

A limitation of this study is that, as a case report, its results may not be generalizable to a broader population and do not allow robust causal inferences. Although the observed improvements were encouraging, these findings should be considered exploratory and require confirmation in further studies before photobiomodulation therapy (PBMT) can be incorporated into standardized supportive care strategies for RRR. Future studies with larger sample sizes are necessary to validate these promising results and to establish evidence-based protocols. In addition, photographic documentation of the irradiated areas is not routinely performed at our institution, which precluded visual comparisons before and after PBMT. Despite these limitations, case reports provide valuable preliminary evidence, particularly in the context of rare conditions such as RRR.

## Conclusion

This scoping review and the case reported suggested that PBMT may be a viable approach for RRR, as it is increasingly being used for the prevention and management of cancer-related toxicities. PBMT may represent a potential safe alternative to corticosteroids and offers a non-invasive, low-cost, non-thermal treatment option with no reported side effects. However, further clinical studies are needed to determine the optimal therapeutic protocol and to conduct long-term follow-up to validate the safety of this approach in oncology patients.

## Patient perspective

The patient expressed her satisfaction with PBMT, reporting significant improvement in symptoms related to RRR and an overall enhancement in quality of life after starting the therapy. She experienced relief from pain associated with RRR. Motivated by her positive experience, the patient independently shared her feedback with others and recommended PBMT to fellow patients undergoing RT at the same hospital.

## Data Availability

The original contributions presented in the study are included in the article/**Supplementary Material**. Further inquiries can be directed to the corresponding author.
