# Supplementary material for: Photobiomodulation therapy approach to a rare phenomenon of radiation recall reaction triggered by cisplatin in a cervical cancer patient: a case report and scoping review
Source: Front Oncol. 2026 Feb 2;15:1700942. doi: 10.3389/fonc.2025.1700942 (PMC12907206; doi:10.3389/fonc.2025.1700942)
Supplement: Supplementary file 1 [file Supplementaryfile1.docx]

**SUPPLEMENTARY MATERIAL**

**Supplementary Material A.**

The search strategy was applied to the databases Cochrane Database via the Cochrane Library, EMBASE via Elsevier, PubMed/MEDLINE via the National Institutes of Health, and Web of Science Core Collection via Clarivate, as well as to the gray literature, including ProQuest™ Dissertation & Theses Citation Index, on March 9, 2025.

| **DATABASES** | **SEARCH STRATEGY** | **RESULTS** |
| --- | --- | --- |
| Cochrane Database via the Cochrane Library | ("radiation recall" OR "RRD" OR "RRR" OR "RRP") AND ("radiotherapy" OR "radiotherapie" OR "radiotherapy s" OR "radiation therapy" OR "irradiated" OR "radiation exposure") AND ("CDDP" OR "cisplatin" OR "platinum-based chemotherapy") in All Text | 4 |
| EMBASE via Elsevier | ('radiation recall'/exp OR 'radiation recall' OR 'rrd' OR 'rrr' OR 'rrp') AND ('radiotherapy'/exp OR 'radiotherapy' OR 'radiotherapie' OR 'radiotherapy s' OR 'radiation therapy'/exp OR 'radiation therapy' OR 'irradiated' OR 'radiation exposure'/exp OR 'radiation exposure') AND ('cddp'/exp OR 'cddp' OR 'cisplatin'/exp OR 'cisplatin' OR 'platinum-based chemotherapy') AND [embase]/lim NOT ([embase]/lim AND [medline]/lim) | 22 |
| **PubMed/MEDLINE via the National Institutes of Health** | ("radiation recall"[All Fields] OR "RRD"[All Fields] OR "RRR"[All Fields] OR "RRP"[All Fields]) AND ("radiotherapy"[MeSH Terms] OR "radiotherapy"[All Fields] OR "radiotherapies"[All Fields] OR "radiotherapy"[MeSH Subheading] OR "radiotherapy s"[All Fields] OR "radiation therapy"[All Fields] OR "irradiated"[All Fields] OR "radiation exposure"[All Fields]) AND ("CDDP"[All Fields] OR "cisplatin"[All Fields] OR "platinum-based chemotherapy"[All Fields]) | 18 |
| **Web of Science Core Collection via Clarivate** | ALL=("radiation recall" OR "RRD" OR "RRR" OR "RRP") AND ALL=("radiotherapy" OR "radiotherapie" OR "radiotherapy s" OR "radiation therapy" OR "irradiated" OR "radiation exposure") AND ALL=("CDDP" OR "cisplatin" OR "platinum-based chemotherapy") | 18 |
| **GREY LITERATURE** | **SEARCH STRATEGY** | **RESULTS** |
| **ProQuest™ Dissertation & Theses Citation Index** | TS=("radiation recall" OR "RRD" OR "RRR" OR "RRP") AND TS=("radiotherapy" OR "radiotherapie" OR "radiotherapy s" OR "radiation therapy" OR "irradiated" OR "radiation exposure") AND TS=("CDDP" OR "cisplatin" OR "platinum-based chemotherapy") | 0 |

**Supplementary Material B.** Excluded articles and the reason why they were excluded (n = 7).

| **Author, year** | **Reason for exclusion** |
| --- | --- |
| Alderman et al. | 4 |
| Antony et al. | 5 |
| Bargagli et al. | 3 |
| Baxevanos et al. | 5 |
| Chauhan et al. | 5 |
| Lemay et al. | 5 |
| Yamada et al. | 3 |

**Reasons for exclusion:**

1. Studies involving patients without cancer (n = 0)
2. Studies involving patients who had not undergone radiotherapy (n = 0)
3. Studies not addressing RRR trigged by cisplatin (n = 2)
4. Studies that did not inform the treatment for RRR (n = 1)
5. reviews, book chapters, expert opinions, and conference abstracts (n = 4)
6. *In vivo* or *in vitro* laboratory studies (n = 0)
7. Not retrieved for full reading (n = 0)

Alderman C, Sargant N, Simpson S, Roy A, Milne A (2013) Radiation recall following cisplatin chemotherapy. Br J Haematol. 163(4): 421. http://doi:10.1111/bjh.12538

Antony R, Fragoso R, Zwienenberg-Lee M, Evans M, Lechpammer M, Ozturk A (2017). RTHP-12. Multi-System Radiation Recall in a Child on Treatment For Medulloblastoma. Neuro-Oncology, 19(6): 221. <https://doi.org/10.1093/neuonc/nox168.896>

Bargagli E, Bonti V, Bindi A, Scotti V, Pistolesi M, Voltolini L, Ferrari K (2018). Fibrotic lung toxicity induced by cytotoxic drugs, radiation and immunotherapy in patients treated for lung cancer. *Monaldi archives for chest disease* *88*(2): 917. <https://doi.org/10.4081/monaldi.2018.917>

Baxevanos P et al (2021) Pneumonitis associated with paclitaxel ifosfamide and cisplatin chemotherapy in a patient with locally advanced penile cancer. Forum of Clinical Oncology 18(2): 29. Available from: http://doi.org/10.2478/fco-2019-0035

Chauhan SL, Chawla M, Lee R (2019) Massive Hemoptysis in a Patient With Gastric Adenocarcinoma: a Case Of Radiation Recall Injury After 40 Years. Elsevier BV 156(4). http://doi.org/10.1016/j.chest.2019.08.2071

Lemay J, Bouchard M, Rousseau E (2020) Radiation Recall Myositis Detected With 18F-FDG PET/CT After a Treatment of Cervical Cancer. Clin Nucl Med. 45(7): e336-e338. http://doi:10.1097/RLU.0000000000003024

Yamada S, Fukugawa Y, Otsuka T, Saito T, Oya N (2024) Radiation-Induced Myonecrosis: a Case Report of a Cervical Cancer Patient With a History of Systemic Lupus Erythematosus. Cureus 16(2): e55134. <http://doi:10.7759/cureus.55134>
